# Extracellular signal-regulated kinases 1/2 as regulators of cardiac hypertrophy

**DOI:** 10.3389/fphar.2015.00149

**Published:** 2015-07-24

**Authors:** Michael Mutlak, Izhak Kehat

**Affiliations:** ^1^The Rappaport Institute and the Bruce Rappaport Faculty of Medicine, Technion - Israel Institute of Technology, Haifa, Israel; ^2^Department of Cardiology and the Clinical Research Institute at Rambam, Rambam Medical Center, Haifa, Israel

**Keywords:** extracellular signal-regulated kinases, mitogen activated protein kinase, cardiac hypertrophy, mouse models, signaling

## Abstract

Cardiac hypertrophy results from increased mechanical load on the heart and through the actions of local and systemic neuro-humoral factors, cytokines and growth factors. These mechanical and neuroendocrine effectors act through stretch, G protein–coupled receptors and tyrosine kinases to induce the activation of a myriad of intracellular signaling pathways including the extracellular signal-regulated kinases 1/2 (ERK1/2). Since most stimuli that provoke myocardial hypertrophy also elicit an acute phosphorylation of the threonine-glutamate-tyrosine (TEY) motif within the activation loops of ERK1 and ERK2 kinases, resulting in their activation, ERKs have long been considered promotors of cardiac hypertrophy. Several mouse models were generated in order to directly understand the causal role of ERK1/2 activation in the heart. These models include direct manipulation of ERK1/2 such as overexpression, mutagenesis or knockout models, manipulations of upstream kinases such as MEK1 and manipulations of the phosphatases that dephosphorylate ERK1/2 such as DUSP6. The emerging understanding from these studies, as will be discussed here, is more complex than originally considered. While there is little doubt that ERK1/2 activation or the lack of it modulates the hypertrophic process or the type of hypertrophy that develops, it appears that not all ERK1/2 activation events are the same. While much has been learned, some questions remain regarding the exact role of ERK1/2 in the heart, the upstream events that result in ERK1/2 activation and the downstream effector in hypertrophy.

## Cardiac Hypertrophy

Countless studies have shown that cardiac hypertrophy is independently associated with an increased risk for heart failure and life threatening arrhythmias. Even with modern treatment the presence of left ventricular hypertrophy in hypertensive patients is associated with a 2.8-fold greater risk for ventricular tachycardia or fibrillation (Chatterjee et al., 2014).

Work overload results in structural remodeling of the heart and in a variety of functional changes (Kehat and Molkentin, 2010b). Pressure overload, such that occurs in patients with hypertension or stenosis of the aortic valve, causes an increase in ventricular mass with a high absolute or relative wall thickness. In these patients both systolic and diastolic ventricular wall stresses are usually normal because concentric hypertrophy counterbalance the effect of elevated systolic and diastolic pressures (Grossman and Paulus, 2013). However, the compensatory increase in ventricular thickness eventually leads to diastolic dysfunction and diastolic heart failure (Gaasch and Zile, 2011).

Eccentric hypertrophy is the result of volume rather than pressure overload on the heart. A typical cause of volume-overload is mitral valve regurgitation, which increases the diastolic volume but not the systolic pressure in the heart. In the initial compensated phase, this hypertrophy corrects systolic ventricular wall stress but fail to normalize diastolic ventricular wall stress (Grossman and Paulus, 2013). Over time the progressive ventricular dilatation grows out of proportion to the myocardial mass, and systolic function deteriorates.

The heart may also undergo growth in response to high-intensity exercise training, termed physiological cardiac hypertrophy. This physiological cardiac hypertrophy is associated with normal or enhanced cardiac function and mostly does not have adverse consequences (Weeks and McMullen, 2011), however, there may be a threshold above which exercise training may also increase the risk of arrhythmias or sudden cardiac death (La Gerche and Prior, 2007). Although eccentric hypertrophy initially resembles physiological growth, and there might be some overlap between these phenomena, its effect on diastolic wall stress and eventual deterioration in function, suggests that maladaptive hypertrophy develops insidiously during volume overload. Patients with systolic heart failure and reduced ejection fraction often, but not always, exhibit ventricular dilatation with eccentric hypertrophy (Gaasch et al., 2008).

At the cardiomyocyte level, cardiac hypertrophy is initiated by alterations in the cell’s architecture and morphology, and cardiomyocytes are able to grow in size through the synthesis and depositions of new sarcomers. Concentric growth elicits a parallel deposition of new sarcomeric units, resulting in transverse growth of the myocytes. In contrast, chronic volume-overload initiates the addition of sarcomeres in series, resulting in a relative increase in cardiomyocyte length. Both experimental evidence and modeling show excellent agreement between changes at the sarcomere, cardiomyocyte, and the whole heart levels (Goktepe et al., 2010). We still do not know precisely how individual myocytes sense the forces imposed by pressure or volume overload and how they are translated in the cell into sarcomerogenesis that results in cell thickening, lengthening, or both. It is also obvious that many functional changes occur in the cardiomyocytes in addition to the structural ones (Kehat and Molkentin, 2010b). The relative importance of the alterations in structure versus change in function is also still unknown.

## Mitogen Activated Protein Kinase (MAPK) Pathway

The mitogen activated protein kinase (MAPK) pathway is a signaling pathway comprised of sequentially acting protein kinases that participate in regulation of cell growth, proliferation, differentiation, survival, transformation, apoptosis and other processes (Shaul and Seger, 2007).

Transmission of signals through this pathway is usually initiated by G protein–coupled receptor, receptor tyrosine kinas or by stress stimuli, and is then carried on by activated small G-proteins, which trigger a cascade of phosphorylation reactions that culminate in the dual phosphorylation and activation of terminal MAPK. These MAPK cascades consist of at least three tiers which constitute the main pathway: MAPK kinase kinase (MAP3K), MAPK kinase (MAP2K), and a MAPK. One or more of the kinase components in each level phosphorylate and activate components in the next level ending with the activation of downstream regulatory proteins by MAPKs and the initiation of the required physiologic effect.

Three main branches of the MAPK signaling pathway have been described and named for their terminal component (MAPK): Extracellular signal-regulated kinases (ERK) 1/2, p38 kinases, and c-Jun N-terminal kinases (JNKs). Human ERK1 and ERK2 have 84% identity in sequence and are believed to share most if not all functions. ERK1 contains additional 17 amino acid addition to the N-terminal domain. Collectively these two proteins (ERK1 and ERK2) are referred to as ERK1/2.

Additional MAP kinases which are not specified to any known pathway have been identified including ERK5, ERK3/4, and ERK8. For each branch, the MAPK component is specifically activated by a distinct MAP2K: MEK1/2 for ERK1/2, MKK3/6 for p38, and MKK4/7 for JNKs. The MAP2K can be activated by more than one MAP3K and it is presumed that this activation is influenced by the type of stimuli. For example, activation of ERK1/2 in response to growth hormone stimulation begins with the MAP3K c-RAF, although pro-inflammatory stimulation could induce activation of ERK1/2 via different MAP3K.

Generally, activation of JNK and p38 kinases is associated more with pro-inflammatory and stress induced signal transduction as opposed to ERK1/2 activation which tends to have greater influence on cell growth and proliferation. Specifically, the p38 cascade regulates angiogenesis, cell proliferation, inflammation and production of cytokines and the JNK cascade plays a role in apoptosis and development of cellular immune system, whereas ERK1/2 are more involved in regulation of meiosis, mitosis and post-mitotic function.

**FIGURE 1:**
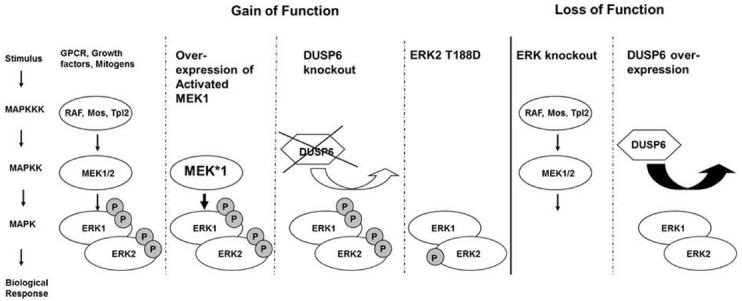
**ERK signaling cascade is classically initiated at the cell membrane, leading to the recruitment and activation of MAP3K, c-RAF.** Downstream of c-RAF are MAPK/ERK kinase (MEK) 1/2 dual-specificity protein kinases which directly activate ERK1/2 by phosphorylation. Gain-of-function approaches included over-expression of a constitutive active MEK1 in the heart, knockout of the phosphatase DUSP6, or overexpression of an ERK2 with a phosphomimetic mutation Threonin 188 to aspartate. Loss of function approaches for ERK included partial or complete knockout of ERK1 and ERK2 from the heart or the over-expression of the cytoplasmic ERK specific phosphatase DUSP6.

In cardiac myocyte, ERK signaling cascade is classically initiated at the cell membrane by activation of the small G protein Ras, leading to the recruitment and activation of MAP3K, c-RAF (Wellbrock et al., 2004). Downstream of c-RAF are MAPK/ERK kinase (MEK) 1/2 dual-specificity protein kinases which are exclusive activators of ERK1/2 (Figure 1). MEK1/2 directly activates ERK1/2 by phosphorylating the threonine-glutamate-tyrosine (TEY) motif located within their activation loops (Shaul and Seger, 2007). Activation of ERK1/2 leads to the phosphorylation of numerous cytoplasmic targets and also to the translocation of ERK into the nucleus and activation of transcription factors such as Elk-1, c-FOS, p53, GATA4, and Ets1/2, which are important regulators of growth and proliferation.

The duration, extent, and subcellular compartment of ERK1/2 activation are important for ERK biological effects. Inactivation of ERK1/2 is mediated mainly by phosphatases such as protein phosphatase 2A (PP2A) or dual-specificity phosphatases (DUSPs) which dephosphorylate either or both regulatory tyrosine and threonine residues in the activation loop (Fjeld et al., 2000). Of the different *Dusp* genes, five have been reported to prefer p38 and JNK over ERK1/2, including DUSP1, DUSP4, DUSP8, DUSP10, and DUSP16. Three cytoplasmic DUSPs: DUSP6, DUSP7, and DUSP9 were reported to be ERK1/2 specific (Dickinson and Keyse, 2006), and one inducible nuclear DUSP, the DUSP5, was shown to be nuclear ERK1/2 specific (Nayak et al., 2014). However, both DUSP7 and DUSP9 may also be able to inactivate p38 (Dickinson and Keyse, 2006).

## MEK-ERK1/2 Gain of Function

Most of the initial research that investigated ERK1/2 in the heart aimed at determining the role of ERK1/2 in cardiac hypertrophy and protection. However, no consensus was achieved in this regard. Studies conducted on cultured neonatal rat cardiomyocytes, which represented the majority of the studies in this field, yielded inconsistent results. Consequently, the use of transgenic and gene-targeted mice has emerged as a more consistent and reliable research methodology.

The first reported experiments using transgenic mice for this application were conducted by Bueno et al. (2000) who generated transgenic mice over-expressing an activated MEK1 mutant under the transcriptional control of cardiac-specific α-myosin heavy chain promoter. Histologic and echocardiographic analysis showed an increase in cardiac wall thickness and improved cardiac function in these mice, with no signs of cardiac decompensation or premature death after a follow-up period of 12 months. It should be noted that overexpression of active MEK1 not only resulted in increased ERK1/2 phosphorylation, but also in increased total ERK1/2 protein levels, presumably through stabilization and reduced degradation of endogenous ERKs. Moreover, these mice were significantly less affected by ischemia/reperfusion induced apoptosis compared with wild type mice (Lips et al., 2004).

To further test the effects of ERK1/2, transgenic mice lines over-expressing ERK2 in the heart were generated. These mice, however, showed no alteration in cardiac size or function compared with wild-type mice indicating that without being activated, ERK2 overexpression is not enough to induce cardiac hypertrophy (Bueno and Molkentin, 2002). This conclusion was supported by results obtained from crossing the MEK1 transgenic mice and the ERK2 transgenic mice which showed synergistic hypertrophy. In line with these observations, transgenic mice over-expressing activated RAS in the heart were generated and showed cardiac hypertrophy. However, they had an increased tendency towards cardiac decompensation and dilatation indicating pathologic response. Activation of additional signaling pathways other than MEK-ERK1/2, by activated RAS could possibly explain this phenotype.

Additional studies conducted on transgenic mice tried to explore alternative, and perhaps more physiologic approaches in achieving ERK1/2 gain of function in the heart. For example, Maillet et al. (2008) explored ERK1/2 gain of function effects on cardiac hypertrophy using *Dusp6* null (^–/–^) mice. These mice showed mild increase in baseline phosphorylation of ERK1/2 compared with wild-type mice indicating higher ERK1/2 baseline activity. However, no increase in the extent or the duration of ERK1/2 activation was observed following hypertrophic stimulation, indicating that DUSP6 specifically regulates basal ERK1/2 level of activity. The *Dusp6* (^–/–^) knockout mice showed significant increase in heart weight without an increase in cardiomyocyte circumference at baseline. Histologic analysis of heart tissue from 3-day-old *Dusp6* ^–/–^ neonates showed a significant increase in the number of proliferating cells indicated by phospho-histone H3 labeling and BrdU incorporation. Consequently, the authors concluded that the increase in cardiac weight is secondary to increased proliferation of cardiomyocytes. Since *Dusp6* knockout did not affect stimulus induced ERK1/2 activation, it was not surprising that stimulus driven hypertrophy using pressure overload or agonists was not different in the *Dusp6* null mice.

Numerous studies in other systems showed that the MEK-ERK pathway is an important regulator of cell proliferation and oncogenic transformation, but outside this study, few ERK1/2 gain-of-function or loss-of-function models documented an effect on cardiomyocyte proliferation. Very recently the role of the NRG1 co-receptor ERBB2 in cardiomyocyte proliferation was examined (D’Uva et al., 2015). Constitutively active ca-ERBB2 transgenic mice showed increased cardiomyocyte proliferation and activation of ERK1/2. The pharmacologic inhibition of MEK-ERK1/2 signaling suppressed the cardiomyocyte proliferation, dedifferentiation, and reduced the hypertrophic response in these ca-ERBB2 transgenic mice, therefore ERK1/2 signaling may be required for cardiomyocyte proliferation.

More recently, a new site of phosphorylation on Threonine 188 (Thr 188) in ERK2 was identified by Lorenz et al. (2009) who introduced an alternative pathway leading to the activation of ERK2 in the heart. In this study, it was shown that stimulation of Gq coupled receptors leads to the release of G protein βγ subunits which serves as scaffold for the RAF-MEK-ERK pathway leading to ERK2 activation; i.e., phosphorylation of the TEY motif, and subsequent ERK2 dimerization and auto-phosphorylation on Thr 188. Furthermore, ERK2 auto-phosphorylation on Thr188 leads to the translocation of ERK2 into the nucleus where it can activate effectors that have been shown to promote cardiac hypertrophy. To study the *in vivo* effect of Thr188 auto-phosphorylation, they generated transgenic mice overexpressing wild-type Erk2 (Erk2 T188T), Erk2 T188A and Erk2 T188S (phosphorylation-deficient Erks), and Erk2 T188D (gain-of-function Erk). Replacing the Threonine at position 188 with the negatively charged aspartate is generally considered to be a phosphomimetic mutation. No hypertrophy was observed in any of these mice compared to wild-type mice at baseline, suggesting that over-expression of wildtype ERK does not lead to hypertrophy, that the phosphorylation on Thr188 on ERK2 is not sufficient to induce cardiac hypertrophy, or that the Erk2T188D mutant dose not mimic the endogenous phosphorylation on Thr188. However, following pressure overload, mice expressing Erk2T188D displayed more prominent hypertrophy than those expressing the wild type or the phosphorylation-deficient ERK2. Furthermore, a follow-up study by Ruppert et al. (2013) concluded that Thr188 phosphorylation is associated with the pathologic form of hypertrophy. They showed that interference with this auto-phosphorylation using the mutant ERK2 T188A, which is dominant-negative for ERKThr188 signaling, attenuated cardiomyocyte hypertrophic responses to phenylephrine and chronic pressure overload *in vitro* and *in vivo*, but did not affect physiological cardiac growth and function (Ruppert et al., 2013).

In summary, these results support a pro-hypertrophic role for MEK1-ERK1/2 signaling, and show that a strong and sustained activation of the cascade is sufficient to cause concentric cardiac hypertrophy with protection against apoptotic stimuli (Figure 1). It also appears that specific mode of ERK1/2 activation may dictate the hypertrophic response of the heart to various stimuli, and that not all ERK1/2 activation events are the same, but the specific role of Thr188 phosphorylation in the heart is not entirely clear.

## ERK1/2 Loss of Function

Loss-of-function approaches were used to assess the necessity of MEK-ERK signaling to cardiac hypertrophy. Due to the lethality of *Erk2* homozygous null mice, initial studies used *Erk1* homozygote null and *Erk2* heterozygote null *Erk1*^–/–^; *Erk2*^+/–^ mice. These mice failed to show a significant reduction in cardiac hypertrophy after pressure overload induced by transverse aortic constriction (TAC) or after exercise-induced cardiac hypertrophy with swimming (Purcell et al., 2007). When crossed to transgenic mice expressing a constitutively active MEK1 transgene, these mice did display less cardiac hypertrophy, demonstrating that ERK1/2 are the MEK proteins targets inducing hypertrophy. Together these results could be taken as evidence that ERK1/2 signaling was not necessary for cardiac hypertrophy, although it was possible that the remaining ERK2 activity was sufficient to mediate the required downstream signaling events. The *Erk2*^+/–^ heterozygous mice did show significant reductions in fractional shortening after TAC compared with wildtype mice and *Erk1*^–/–^ mice showed a trend toward reduced function, showing that while ERK1/2 activity may not be necessary for concentric hypertrophy, it does play a role in the process.

Dual-specificity MAP kinase phosphatases (MKPs) provide a negative regulatory network on MAPK activation. Of those, DUSP6 was shown to specifically bind ERK1 and ERK2 *in vitro*, and this specific binding allows it to specifically dephosphorylate and inactivate these MAPKs *in vivo* in the cytoplasm (Caunt and Keyse, 2013). Several lines of transgenic mice overexpressing DUSP6 specifically in the heart were generated, and as expected did not demonstrate evidence for cardiac ERK1/2 phosphorylation even after stimulation. Similar to the *Erk1*^–/–^ and *Erk2*^+/–^ null mice the DUSP6 overexpressing mice showed the same degree of hypertrophy after TAC pressure overload or after swimming as wildtype mice (Purcell et al., 2007). Following long term pressure overload of 14 weeks after TAC the DUSP6 overexpressing mice did show more cardiac hypertrophy of the eccentric type with dilation, inflammation, and reduced function. This model also supported the notion that ERK1/2 signaling was not necessary for concentric hypertrophy, but did suggest for the first time that EKR1/2 activity may be required to prevent eccentric growth in the context of pressure-overload. It should be noted though, that while DUSP6 specifically inactivate ERK1/2 in the cytoplasm, DUSP5 is responsible for dephosphorylating nuclear ERK. Therefore, it could not be completely ruled out that some nuclear ERK activity was still present in DUSP6 transgenic mice hearts.

To more fully understand the necessity of ERK1/2 signaling for cardiac hypertrophy we generated cardiac specific ERK1/2 targeted mice: Erk1^–/–^; Erk2 fl/fl and used two different Cre recombinase lines- the Nkx2.5 knock-in Cre or the α-Myosin heavy chain transgenic Cre to eliminate both ERK1 and ERK2 from cardiomyocytes in the heart (Kehat et al., 2011). Elimination of either ERK1 alone in ERK1^–/–^ mice or the elimination of cardiac ERK2 alone in *ERK2 fl/fl;Cre* mice did not result in a baseline phenotype. However, the elimination of both from the heart resulted in spontaneous eccentric hypertrophy with dilatation of the heart and elongation of the cardiomyocytes. As predicted by Laplace equation the dilation of the heart resulted in increased diastolic wall stress with eventual reduced function, further cardiac dilatation and ultimately death. To explore the mechanism of reduced function in these mice we also studied the elongated cardiomyocytes after isolation. While the function of the dilated heart was reduced, the function of the elongated cardiomyocytes was increased, further demonstrating that the increase diastolic wall stress was responsible for the reduced function of the heart.

Cardiomyocytes from cardiac ERK1/2 null mice (Erk1^–/–^; Erk2 fl/fl;Cre) displayed spontaneous elongation suggesting that ERK1/2 signaling is required to inhibit eccentric growth of the heart. When stressed with stimuli that elicit concentric growth like angiotensin II and adrenergic phenylephrine administration these cardiomyocytes responded with growth both in width and in length. In agreement with other models, these data show that ERK1/2 signaling may be sufficient but is not required to myocyte growth in width and concentric growth of the heart. Yet, ERK1/2 signaling is required to prevent myocyte elongation and eccentric growth both at baseline and during stimuli that usually cause concentric growth (Figure 1).

Fibrosis with increased collagen accumulation between myocytes is a structural hallmark of prolonged pressure overload and concentric hypertrophy, and to a lesser degree may accompany volume overload (Spinale, 2007). However, neither the activated MEK1 transgenic mice nor the Erk1^–/–^; Erk2 fl/fl;Cre null mice showed any increase in collagen accumulation even after long term follow up for the former and with deteriorating function in the latter. This dissociation of cardiomyocyte growth from the fibrosis that is usually associated with it suggests that ERK1/2 signaling may be specifically dedicated to control the structural aspect of the cardiomyocyte hypertrophy.

## Pharmacologic Agents to Study ERK1/2 Loss of Function

ERKs have long been implicated as regulators of cardiac hypertrophy on account of their activation in response to most, if not all, stress stimuli known to induce hypertrophic growth. As a result, intense efforts are underway to develop compounds that inhibit this pathway and thus provide a potential therapeutic agent that could attenuate cardiac growth. In addition, numerous studies in the pathogenesis of cancer are exploring these MEK inhibitors in attempt to develop therapeutic agents for the treatment of cancer.

The most studied inhibitors of the MEK1/2-ERK1/2 pathway are U0126 and PD98056. These inhibitors do not target the ATP binding pocket of MEK and therefore are relatively MEK specific (Duncia et al., 1998; English and Cobb, 2002). However, they do inhibit all MEKs including MEK1, MEK2 and to a lesser extent MEK5.

Several *in vitro* studies have explored these MEK inhibitor’s effects in cultured cardiomyocytes. MEK inhibition using U0126 attenuated contractile dysfunction and abolished the increase in ANF gene expression induced by endothelin-1 and isoprenaline (Munzel et al., 2005). U0126 also blocked cardiomyocyte growth induced by endothelin-1 and phenylephrine (Yue et al., 2000). Likewise, PD98059 reduced b-type natriuretic promotor activity induced by endothelin or mechanical strain (Liang et al., 2000) and reversed leukemia inhibitory factor (LIF)-induced cardiomyocyte hypertrophy (Kodama et al., 2000). Collectively these studies support the pro-hypertrophic effects of the MEK-ERK cascade.

The effects of pharmacologic inhibitors of ERK1/2 have been demonstrated *in vivo* as well. It has been shown that the NO synthase inhibitor (L-NAME) induced myocardial remodeling was markedly attenuated by oral administration of PD98059 in Wistar-Kyoto rats (Sanada et al., 2003). Additionally, a study that explored the effects of the regulator of G protein signaling 5 (Rgs5) on cardiac remodeling and heart failure showed a protective role of Rgs5 in the presence of pressure overload which was attributed to the inhibition of MEK1/2-ERK1/2 pathway (Li et al., 2010). Indeed, cardiac dilatation and decompensation, induced by pressure overload, were much more prominent in Rgs5^–/–^ mice compared with Rsg5^+/+^ mice and were markedly reversed when these mice were treated with U0126. Exercise results in physiologic cardiac hypertrophy, and voluntary running was also shown to result in activation of the ERK cascade. Treatment with U0126 in this setting did not prevent the cardiac hypertrophy but did reduce the cytoplasmic translocation of PPARα (el Azzouzi et al., 2012). The inhibitory effect on PPARα was shown to be mediated by MEK1, and not by its downstream effector kinase ERK1/2 (el Azzouzi et al., 2012). These studies also indicate that MEK may be necessary for cardiac hypertrophy. However, as with all other pharmacological approaches, it is very difficult to completely exclude off-target effects of these drugs, as in particular some effects on MEK5 have been noted.

## ERK1/2 Activation Patterns

**FIGURE 2:**
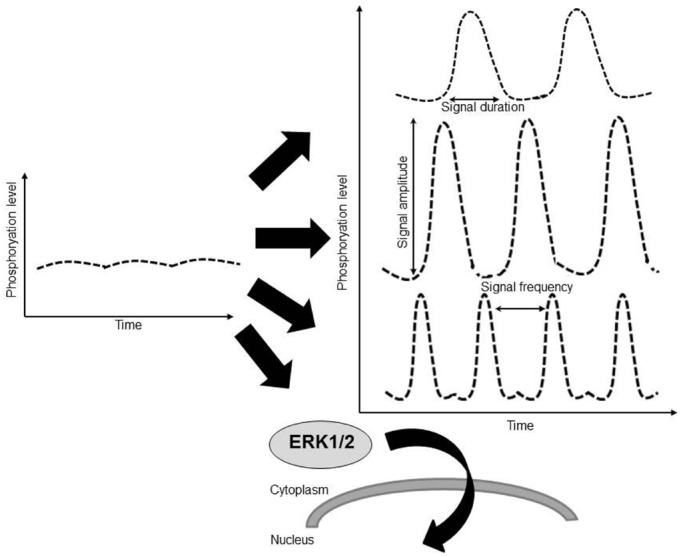
**The quantitative information about a constant extracellular stimulus like pressure overload may be carried by the duration of ERK activation, by amplitude of ERK activation (phosphorylation level), by the frequency with which the activity of ERK shifts between active and inactive states, by the translocation of ERK to a subcellular location such as the nucleus, or through a combination of these factors**.

The quantitative information about a constant extracellular stimulus like pressure overload may be carried by the number of active ERK molecules, or signal amplitude, or by the frequency with which the activity of ERK shifts between active and inactive states, or signal frequency (Figure 2). It is also not clear whether the cell responds to the absolute number of phosphorylated ERK molecules or to the fraction of phospho-ERK to total ERK molecules in the cytoplasm or nucleus. For example, the interpretation of constant epidermal growth factor receptor signaling in cells was shown to result in discrete pulses of ERK activation and incorporated both frequency and amplitude modulated elements (Albeck et al., 2013). A study in human cancer cells found a very large cell to cell variability in the levels of nuclear ERK2. Despite this variability, the amount of ERK2 entering the nucleus upon EGF stimulation was proportional to the basal level of nuclear ERK2 in each cell, suggesting a fold-change response mechanism (Cohen-Saidon et al., 2009). Therefore the question whether downstream targets of ERK respond to ERK fold or absolute changes in phospho-ERK molecules is still open. It should be noted that overexpression of wild-type ERK in the heart did not result in cardiac hypertrophy both at baseline or following TAC pressure overload and that overexpression of constitutively active mutant of MEK1 in the heart resulted in an increase in both total ERK1/2 and phospho-ERK1/2. Since a detailed estimation of phospho-ERK to total ERK ratios in both nucleus and cytoplasm was not performed in these studies it is difficult to draw direct conclusion from these studies regarding ERK cellular sensing.

It is also not understood how pressure and volume overload are interpreted as ERK activity in the heart. Evidence from cultured cells suggests that ERK activity occurs in bursts of about 30 min, and strong stimuli cause an increase in pulse duration and a decrease in the spacing between pulses (Albeck et al., 2013). Due to this fast kinetic it is virtually impossible to assess the relationship between pressure or volume overload and the associated profile of ERK1/2 activation *in vivo* over time with current tools. Acutely, most hypertrophic stimuli appear to activate and induce phosphorylation of ERK1/2 (Kehat and Molkentin, 2010a). Comparison between TAC pressure overload model and vena cava fistula volume overload model showed ERK1/2 activation at 24 h with the former but not with the latter, hinting at different oscillatory behavior of ERK1/2 activation in pressure and in volume overload (Lorenz et al., 2009). Genetic manipulation of ERK1/2 activity in mice hearts allows precise and robust modulation of the cascade. However, these manipulations only mimic the extreme ends of the physiologic activity spectrum where activity is either extremely strong or completely lacking. Therefore the exact pathophysiological connections between mechanical overload, patterns of ERK activation, and growth patterns of the heart are still not clear.

## ERK1/2 Targets in Hypertrophy

Several proteins have been suggested as the downstream ERK1/2 targets that mediate hypertrophy. ERK1/2 directly phosphorylate the transcriptional effectors Elk-1, Ets1, Sap1a, and c-Myc

(Bueno and Molkentin, 2002). In cultured cardiomyocytes the phosphorylation of Elk-1 by ERK was shown to be important for early gene activation following phenylephrine-induced hypertrophy (Babu et al., 2000), and c-Myc is a driver of cardiac hypertrophy (Zhong et al., 2006). Mitogen- and stress-activated protein kinase-1 (MSK1) is another ERK1/2 target that was implicated in phenylephrine induced cardiomyocyte hypertrophy (Markou et al., 2004).

The transcription factor GATA4 is an important regulator of stimuli induced cardiac hypertrophy. Knock-in mice in which Serine 105 was replaced with alanine in GATA4 failed to develop cardiac hypertrophy in response to pressure overload or adrenergic stimulation with phenylephrine, suggesting that phosphorylation of GATA4 in this position is crucial for GATA4 function (van Berlo et al., 2011). GATA4 can be directly phosphorylated at Serine 105 by ERK1/2 and p38, and both ERK1/2 and p38 activity were shown to be necessary for the increase in GATA4 DNA binding that occurred following acute wall stretching (Tenhunen et al., 2004). Activated MEK1 transgenic mice failed to develop hypertrophy when crossed to GATA4 null mice (Gata4 fl/fl; β-Myosin Heavy chain Cre), showing that GATA4 is required for MEK-ERK1/2 hypetrophic function. When the activated MEK1 transgenic mice were crossed to the GATA4 S105A mutant mice cardiac hypertrophy was significantly blunted, suggesting that phosphorylation of GATA4 at Serine 105 is required for MEK-ERK1/2 hypetrophic function.

Potential ERK1/2 targets were also assessed in order to understand the function of Thr188 ERK2 phosphorylation (Lorenz et al., 2009). The basal level of phosphorylation of the Erk1/2 targets p90RSK, p70S6K, Elk1, MSK1, and c-Myc was similar in all Erk2T188-mutant mouse lines examined suggested that Thr188 phosphorylation is not sufficient to activate ERK1/2, or that the phosphomimetic mutation did not fully mimic phosphorylation at Thr188 position. Induction of pressure overload by TAC also increased the level of phosphorylation of the cytosolic proteins p90RSK and p70S6K similarly in all the mice lines. In contrast the phosphorylation levels of the nuclear Elk1, MSK1, and c-Myc following TAC pressure overload was higher in the Erk2 T188D phosphomimetic mutant hearts. These data suggested that Thr188 phosphorylation in combination with pressure overload stimulation preferentially increased the phosphorylation of nuclear ERK1/2 targets. It was also suggested that hypertrophic stimuli induce nuclear ERK1/2 signaling by suppressing expression of DUSP5 through MEK-ERK1/2 and class I HDAC-dependent mechanism, and that class I HDAC inhibition blocks nuclear ERK1/2 activation (Ferguson et al., 2013). Overexpression of DUSP5 inhibited agonist induced hypertrophy in cardiomyocytes strengthening the view that nuclear ERK1/2 activity is important for hypertrophy.

## Summary

The many studies described leave little doubt that MEK-ERK1/2 signaling is important for cardiac hypertrophy. Stimuli that elicit concentric hypertrophy such as pressure overload and Gq coupled receptor activation result in activation of the cascade at least acutely, and gain-of-function models showed increased hypertrophy. In contrast loss-of-function model showed cardiac dilatation and eccentric hypertrophy. It is also clear that not all ERK1/2 activation events are the same. Sustained baseline activation, post-stimulus peak activation or activation amplitude, the duration or frequency of activation, and the cytoplasmic versus the nuclear localization of activated ERK all likely play different roles. Carful dissection of these activation events in the future may reveal more refined roles for ERK activation and cardiac hypertrophy.

### Conflict of Interest Statement

The authors declare that the research was conducted in the absence of any commercial or financial relationships that could be construed as a potential conflict of interest.
